# Upregulation of Neogenin-1 by a CREB1-BAF47 Complex in Vascular Endothelial Cells is Implicated in Atherogenesis

**DOI:** 10.3389/fcell.2022.803029

**Published:** 2022-02-03

**Authors:** Nan Li, Hong Liu, Yujia Xue, Junliang Chen, Xiaocen Kong, Yuanyuan Zhang

**Affiliations:** ^1^ Department of Human Anatomy, Nanjing Medical University, Nanjing, China; ^2^ Key Laboratory of Targeted Intervention of Cardiovascular Disease and Collaborative Innovation Center for Cardiovascular Translational Medicine, Nanjing Medical University, Nanjing, China; ^3^ Department of Pathophysiology, Wuxi Medical School, Jiangnan University, Wuxi, China; ^4^ Department of Endocrinology, Nanjing First Hospital, Nanjing Medical University, Nanjing, China; ^5^ Institute of Biomedical Research, Liaocheng Univeristy, Liaocheng, China; ^6^ Hainan Provincial Key Laboratory for Tropical Cardiovascular Diseases Research, Key Laboratory of Emergency and Trauma of Ministry of Education, Department of Cardiology, The First Affiliated Hospital of Hainan Medical University, Haikou, China

**Keywords:** transcriptional regulation, endothelial cell, chromatin remodeling protein, atherosclerosis, transcription factor

## Abstract

Atherosclerosis is generally considered a human pathology of chronic inflammation, to which endothelial dysfunction plays an important role. Here we investigated the role of neogenin 1 (Neo-1) in oxidized low-density lipoprotein (oxLDL) induced endothelial dysfunction focusing on its transcriptional regulation. We report that Neo-1 expression was upregulated by oxLDL in both immortalized vascular endothelial cells and primary aortic endothelial cells. Neo-1 knockdown attenuated whereas Neo-1 over-expression enhanced oxLDL-induced leukocyte adhesion to endothelial cells. Neo-1 regulated endothelial-leukocyte interaction by modulating nuclear factor kappa B (NF-κB) activity to alter the expression of adhesion molecules. Neo-1 blockade with a blocking antibody ameliorated atherogenesis in *Apoe* ^−/−^ mice fed a Western diet. Ingenuity pathway analysis combined with validation assays confirmed that cAMP response element binding protein 1 (CREB1) and Brg1-associated factor 47 (BAF47) mediated oxLDL induced Neo-1 upregulation. CREB1 interacted with BAF47 and recruited BAF47 to the proximal Neo-1 promoter leading to Neo-1 trans-activation. In conclusion, our data delineate a novel transcriptional mechanism underlying Neo-1 activation in vascular endothelial cells that might contribute to endothelial dysfunction and atherosclerosis.

## Introduction

Coronary heart disease represents one of the major causes for heart failure, which affects ∼30 million patients annually and is the leading cause of deaths worldwide (Savarese and Lund, 2017). Atherosclerosis is characterized by the deposition of fat-laden plaques in the arteries causing progressive narrowing of the blood vessel and subsequently coronary heart disease (Libby, 2021a). Decades of research have led to the consensus that atherosclerosis is a human pathology of chronic vascular inflammation (Libby, 2021b). On the one hand, multiple different populations of immune cells, including macrophages, granulocytes, lymphocytes, and natural killer cells, are present in the atherosclerotic plaque in a stage-dependent manner (Libby and Hansson, 2015). On the other hand, modulating the inflammatory response in the vessels has been shown to alter the development and progression of atherosclerosis in model animals (Libby et al., 2013). The most convincing piece of evidence to support the long-held view that vascular inflammation is the linchpin of atherogenesis comes from a recently published clinic study that shows the efficacy of a monoclonal antibody targeting the pro-inflammatory cytokine IL-1β (Canakinumab) in the treatment of atherosclerosis (Ridker et al., 2017).

Hyperlipidemia, or the presence of elevated levels of oxidized low-density lipoprotein (oxLDL) in the circulation, is a major risk factor for atherosclerosis (Steinberg and Witztum, 2010). High levels of oxLDL contribute to atherosclerosis by skewing the phenotypes of vascular cells including endothelial cells. Endothelial dysfunction is considered one of the early pathophysiological events during atherogenesis (Gimbrone and Garcia-Cardena, 2016). Under physiological conditions, the circulating leukocytes (e.g., macrophages) are unable for firmly attach to the vessel wall. In the presence of oxLDL, however, endothelial cells upregulate the expression of a plethora of different adhesion molecules including intercellular adhesion molecules (ICAMs), vascular adhesion molecules (VCAMs), and selectins that mediate the rolling, adhesion, and penetration of leukocytes (Blankenberg et al., 2003). Indeed, blocking antibodies that targets either adhesion molecules directly or their receptors expressed on the surface of leukocytes have been shown to reduce atherogenesis with high efficiency in mice (Galkina and Ley, 2007). Transcriptionally, nuclear factor kappa B (NF-κB) is considered a master regulator of adhesion molecules. Conserved NF-κB motifs have been identified on the promoters of adhesion molecule genes (Tak and Firestein, 2001). Consistently, manipulation of NF-κB activity in endothelial cells, through deletion of its upstream activator IKKγ or over-expression of its upstream inhibitor IκB, protects the mice from Western diet feeding induced atherosclerosis with concomitant down-regulation of adhesion molecules and reduced leukocyte adhesion (Gareus et al., 2008).

Neogenin 1 (Neo-1) was originally identified as a receptor for netrins, a group of guidance molecules; engagement of Neo-1 by netrins provides the cue for neurons to expand and form connections (Livesey, 1999). Recent investigations have expanded the realm of pathophysiological events regulated by Neo-1. A string of reports have suggested that Neo-1 may play a role in regulating the inflammatory response in different tissues. Genetic ablation or pharmaceutical inhibition of Neo-1 in mice can lead to attenuation of ischemia-reperfusion induced hepatic inflammation, zymosan A induced peritonitis, and high pressure ventilation induced pulmonary inflammation (Konig et al., 2012; Mirakaj et al., 2012; Schlegel et al., 2014). Based on these prior observations, we hypothesized that Neo-1 might be involved in atherogenesis by regulating vascular inflammation. We report here that oxLDL upregulates Neo-1 in vascular endothelial cells at the transcriptional level via the CREB1-BAF47 complex. Neo-1 promotes leukocyte adhesion to endothelial cells by modulating NF-κB dependent trans-activation of adhesion molecules. Importantly, Neo-1 inhibition in *Apoe* ^−/−^ mice dampens atherosclerosis.

## Methods

### Animals

All animal protocols were reviewed and approved by the intramural Ethics Committee on Humane Treatment of Laboratory Animals of Nanjing Medical University. The mice were maintained in an SPF environment with 12 h light/dark cycles and *ad libitum* access to food and water. To induce atherosclerosis, 8-wk male *Apoe* ^−/−^ mice were fed a Western diet (D12109, Research Diets, New Brunswick, NJ, United States) for 8 weeks as previously described (Zhang et al., 2020). An anti-Neo1 antibody (5 μg per injection, R&D, AF 1079) or isotype IgG was injected intravenously every day from week 5 of Western diet feeding till the mice were sacrificed. The animals were euthanized by pentobarbital sodium (100–120 mg/kg) to obtain their samples. Atherosclerotic lesions were gauged by en face analysis of the whole aorta and by cross-sectional analysis of the proximal aorta essentially described previously (Liao et al., 2020; Huangfu et al., 2021).

### Cell Culture, Plasmids, Transient Transfection, and Reporter Assay

Human immortalized umbilical vein endothelial cells (HUVEC/EAhy926, ATCC), human monocytic/macrophage cells (THP-1, ATCC), and human embryonic kidney cells (HEK293, Invitrogen) were maintained in DMEM (Invitrogen) supplemented with 10% fetal bovine serum (FBS, Hyclone) as previously described (Chen et al., 2020; Yang et al., 2021). Human primary aortic endothelial cells (HAEC, Cambrex/Lonza) were maintained in EGM-2 media with supplements supplied by the vendor; experiments were performed in primary cells between 3rd and 6th passages as previously described (Li et al., 2020). FLAG-tagged CREB1 (Mayr et al., 2005) and GFP-tagged BAF47 (Kadoch and Crabtree, 2013) have been described previously. Neo-1 promoter-luciferase construct was made by amplifying genomic DNA spanning the proximal promoter and the first exon of Neo-1 gene (−1274/+101) and ligating into a pGL3-basic vector (Promega). Truncation mutants were made using a QuikChange kit (Thermo Fisher Scientific, Waltham, MA, United States) and verified by direct sequencing. Small interfering RNAs were purchased from Dharmacon. Transient transfection was performed with Lipofectamine 2000. Cells were harvested 48 h after transfection and reporter activity was measured using a luciferase reporter assay system (Promega) as previously described (Liu et al., 2021).

### Protein Extraction, Immunoprecipitation and Western Blot

Whole cell lysates were obtained by re-suspending cell pellets in RIPA buffer (50 mM Tris pH7.4, 150 mM NaCl, 1% Triton X-100) with freshly added protease inhibitor (Roche) as previously described (Lv et al., 2021). Specific antibodies or pre-immune IgGs were added to and incubated with cell lysates overnight before being absorbed by Protein A/G-plus Agarose beads (Santa Cruz). Precipitated immune complex was released by boiling with 1X SDS electrophoresis sample buffer. Western blot analyses were performed with anti-Neo1 (Abcam, ab183511, 1:1000), anti-CREB1 (Proteintech, 12208-1, 1:1000), anti-BAF47 (Proteintech, 20654-1, 1:1000), anti-FLAG (Sigma, F1804, 1:5000), anti-GFP (Proteintech, 50430-2, 1:1000), and anti-β-actin (Sigma, A2228, 1:4000) antibodies.

### Chromatin Immunoprecipitation

Chromatin Immunoprecipitation (ChIP) assays were performed essentially as described before (Coarfa et al., 2020; Maity et al., 2020; Wang J.-N. et al., 2020; Wang S. et al., 2020; Marti et al., 2021). In brief, chromatin in control and treated cells were cross-linked with 1% formaldehyde. Cells were incubated in lysis buffer (150 mM NaCl, 25 mM Tris pH 7.5, 1% Triton X-100, 0.1% SDS, 0.5% deoxycholate) supplemented with protease inhibitor tablet and PMSF. DNA was fragmented into ∼200 bp pieces using a Branson 250 sonicator. Aliquots of lysates containing 200 μg of protein were used for each immunoprecipitation reaction with anti-NF-κB/p65 (Santa Cruz, sc-372), anti-CREB1 (Millipore, 17-600), anti-BAF47 (Cell Signaling Tech, 91735), or pre-immune IgG. For re-ChIP, immune complexes were eluted with the elution buffer (1% SDS, 100 mM NaCO_3_), diluted with the re-ChIP buffer (1% Triton X-100, 2 mM EDTA, 150 mM NaCl, 20 mM Tris pH 8.1), and subject to immunoprecipitation with a second antibody of interest.

### RNA Isolation and Real-Time PCR

RNA was extracted with the RNeasy RNA isolation kit (Qiagen). Reverse transcriptase reactions were performed using a SuperScript First-strand Synthesis System (Invitrogen) as previously described (Hong et al., 2020). Real-time PCR reactions were performed on an ABI Prism 7500 system with the following primers: human *NEO1*, 5′-GGA​GCC​GGT​GGA​TAC​ACT​CT-3′ and 5′-TGG​CGT​CGA​TCA​TCT​GAT​ACT​A-3′; human *ICAM1*, 5′-ATG​CCC​AGA​CAT​CTG​TGT​CC-3′ and 5′-GGG​GTC​TCT​ATG​CCC​AAC​AA-3′; human *VCAM1*, 5′-GGG​AAG​ATG​GTC​GTG​ATC​CTT-3′ and 5′-TCT​GGG​GTG​GTC​TCG​ATT​TTA-3′; mouse *Icam1*, 5′-GTG​ATG​CTC​AGG​TAT​CCA​TCC​A-3′ and 5′-CAC​AGT​TCT​CAA​AGC​ACA​GCG-3’; mouse *Icam2*, 5′-ATG​GTC​CGA​GAA​GCA​GAT​AGT-3′ and 5′-TGC​TGT​TGA​ACG​TGG​CTG​T-3’; mouse *Vcam1*, 5′-TTG​GGA​GCC​TCA​ACG​GTA​CT-3′ and 5′-GCA​ATC​GTT​TTG​TAT​TCA​GGG​GA-3’; mouse *Il1b*, 5′-GAA​ATG​CCA​CCT​TTT​GAC​AGT​G-3′ and 5′-TGG​ATG​CTC​TCA​TCA​GGA​CAG-3’; mouse *Il6*, 5′-TGG​GGC​TCT​TCA​AAA​GCT​CC-3′ and 5′-AGG​AAC​TAT​CAC​CGG​ATC​TTC​AA-3’; mouse *Tnfa*, 5′-CTG​GAT​GTC​AAT​CAA​CAA​TGG​GA-3′ and 5′-ACT​AGG​GTG​TGA​GTG​TTT​TCT​GT-3′; mouse *Infg*, 5′-TCC​TCG​CCA​GAC​TCG​TTT​TC-3′ and 5′-ACG​GCT​CCC​AAG​TTA​GAA​TCT-3’; mouse *Mcp1*, 5′-AAA​ACA​CGG​GAC​GAG​AAA​CCC-3′ and 5′-ACG​GGA​ACC​TTT​ATT​AAC​CCC​T-3’; mouse *Rantes*, 5′-GCT​GCT​TTG​CCT​ACC​TCT​CC-3′ and 5′-TCG​AGT​GAC​AAA​CAC​GAC​TGC-3’. Ct values of target genes were normalized to the Ct values of housekeeping control gene (18s rRNA, 5′-CGC​GGT​TCT​ATT​TTG​TTG​GT-3′ and 5′-TCG​TCT​TCG​AAA​CTC​CGA​CT-3′ for both human and mouse genes) using the ΔΔCT method and expressed as relative mRNA expression levels compared to the control group which is arbitrarily set as 1.

### Leukocyte Adhesion Assay

Leukocyte adhesion assay was performed as previously described. Briefly, THP-1 cells were stained with a fluorescent die (2′,7′-Bis-(2-carboxyethyl)-5(6)-carboxyfluorescein tetrakis (acetoxymethyl) ester) (Sigma) for 30 min at 37°C. After several washes with PBS, THP-1 cells were co-incubated for 30 min with endothelial cells. Unbound leukocytes were removed by washing and the number of adhered cells was visualized by fluorescence microscopy and analyzed with Image-Pro Plus (Media Cybernetics). For each group, at least six different fields were randomly chosen and the positively stained cells were counted and divided by the number of total cells. The data are expressed as relative EdU staining compared to the control group arbitrarily set as 1.

### Statistical Analysis

One-way ANOVA with post-hoc Scheff´e analyses were performed by SPSS software (IBM SPSS v18.0, Chicago, IL, United States). Unless otherwise specified, values of *p* < .05 were considered statistically significant.

## Results

### Neogenin 1 Expression is Upregulated by Oxidized Low-Density Lipoprotein in Endothelial Cells

**FIGURE 1:**
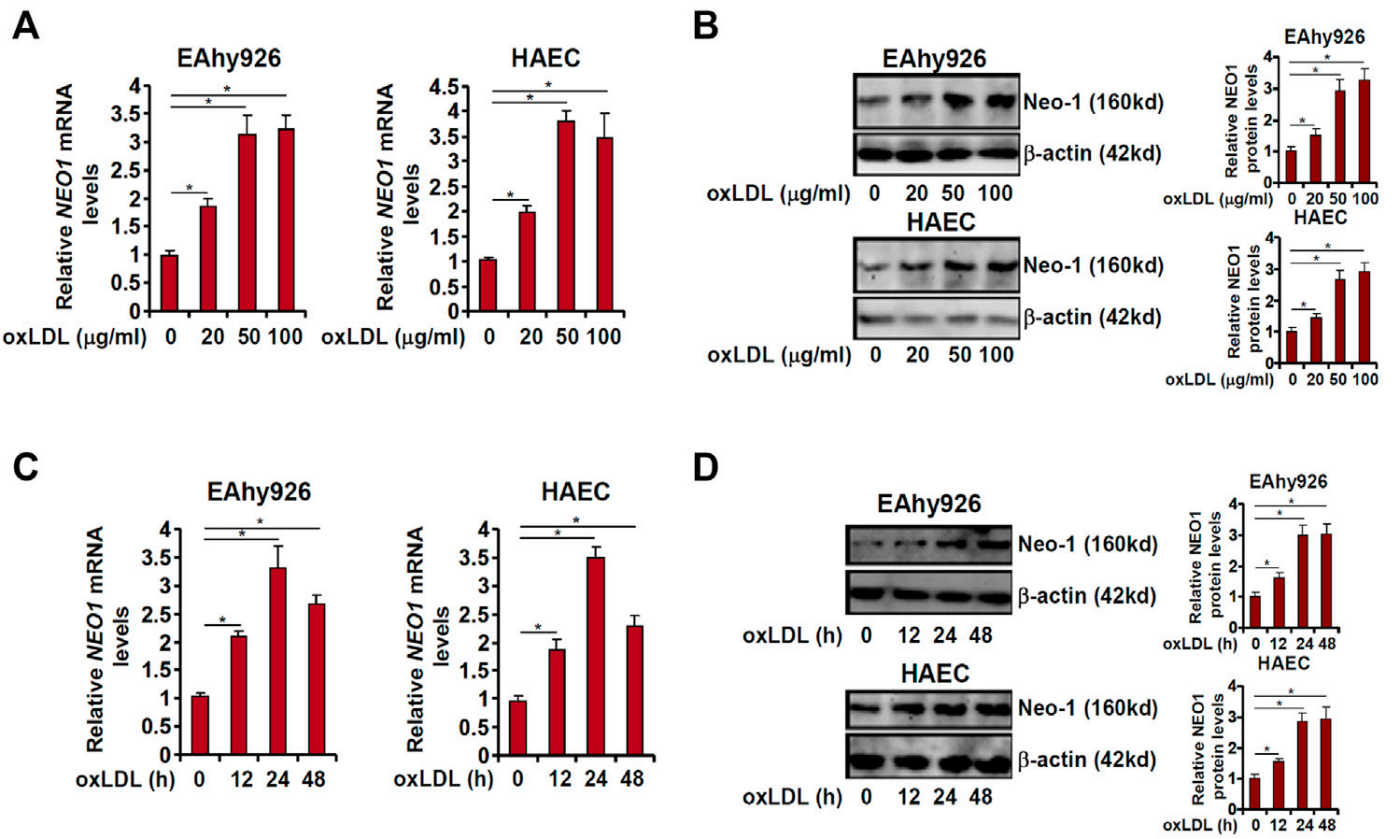
Neo-1 expression is upregulated by oxLDL in endothelial cells. **(A,B)** EAhy926 and HAECs were treated with different concentrations of oxLDL for 24 h. Neo-1 expression was examined by qPCR and Western. **(C,D)** EAhy926 and HAECs were treated with oxLDL (50 μg/ml) and harvested at indicated time points. Neo-1 expression was examined by qPCR and Western.

In order to determine the effect of pro-atherosclerotic stimuli on Neo-1 expression, immortalized human vascular endothelial cells (EAhy926) and primary human aortic endothelial cells (HAECs) were treated with different doses of oxidized low-density lipoprotein (oxLDL). As shown in Figure 1A, oxLDL at 20 μg/ml upregulated Neo-1 message RNA levels by ∼2xfold in both EAhy926 and HAECs as measured by quantitative PCR whereas oxLDL at 50 μg/ml and 100 μg/ml comparably increased Neo-1 mRNA levels by more than 3xfold. Western blotting confirmed that Neo-1 protein levels were similarly upregulated by oxLDL treatment in a dose-dependent manner (Figure 1B). Next, a time course experiment was performed in which the cells were treated with 50 μg/ml oxLDL for different periods of time. QPCR analysis showed that Neo-1 mRNA peaked at 24 h but declined at 48 h followed oxLDL stimulation whereas Western blotting showed that changes of Neo-1 protein levels lagged those of Neo-1 mRNA levels (Figures 1C,D). Of note, Western blotting showed that Neo-1 was exclusively located to the cell membrane and that oxLDL stimulation did not appear to influence its sub-cellular localization (Supplementary Figure S1).

### Neogenin 1 Regulates Leukocyte Adhesion

**FIGURE 2:**
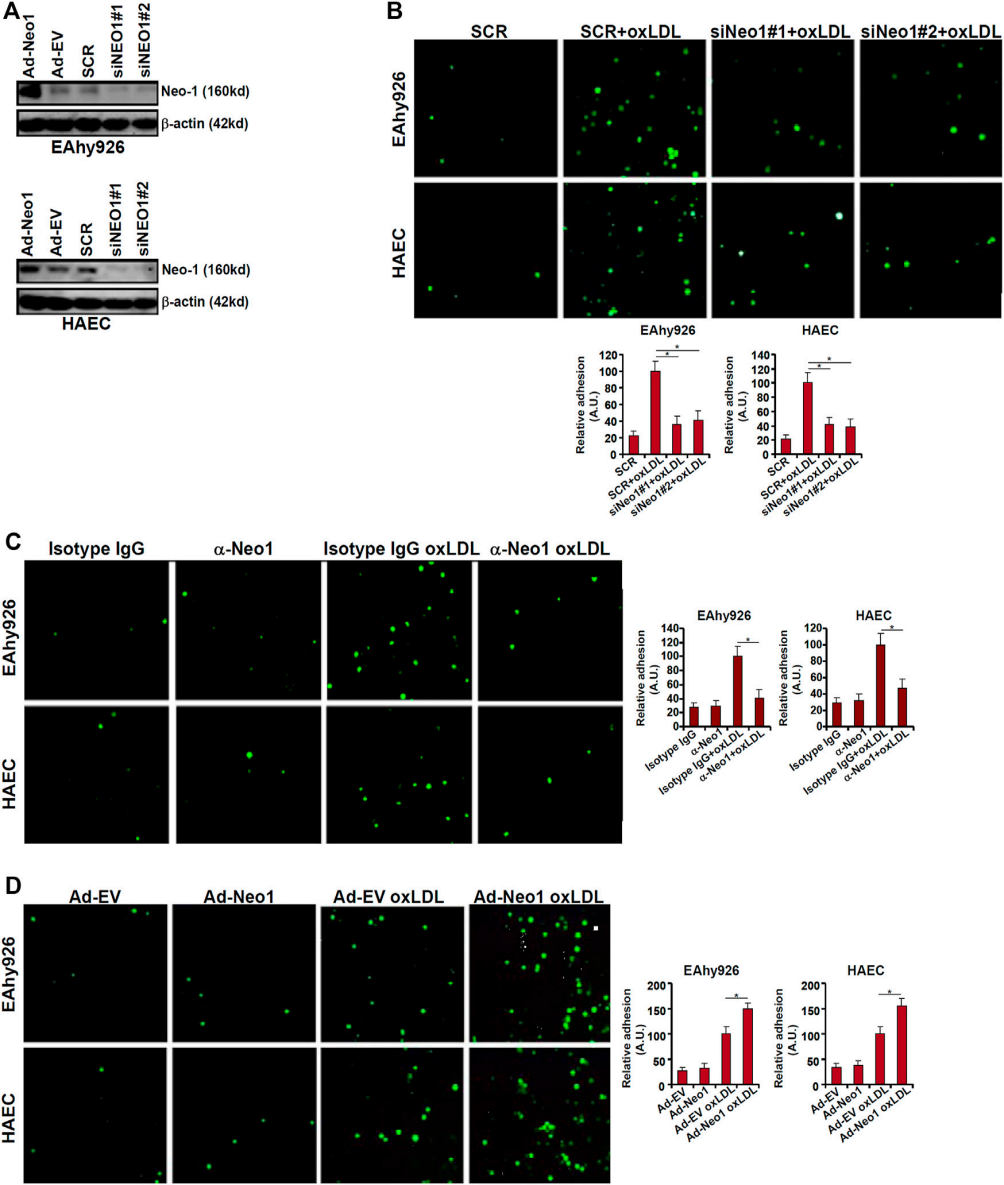
Neo-1 regulates leukocyte adhesion. **(A)** EAhy926 cells and HAECs were transfected with indicated siRNAs. Alternatively, these cells were transduced with adenovirus carrying a Neo-1 vector (Ad-Neo1) or control adenovirus (Ad-EV). Neo-1 protein levels were examined by Western. **(B)** EAhy926 cells and HAECs were transfected with indicated siRNAs followed by treatment with oxLDL (50 μg/ml) for 24 h. Leukocyte adhesion was performed as described in Methods. **(C)** EAhy926 cells and HAECs were treated with oxLDL (50 μg/ml) in the presence or absence of an anti-Neo-1 antibody for 24 h. **(D)** EAhy926 cells and HAECs were transduced with adenovirus carrying a Neo-1 vector (Ad-Neo1) or control adenovirus (Ad-EV) followed by treatment with oxLDL (50 μg/ml) for 24 h. Leukocyte adhesion was performed as described in Methods. SCR, scrambled siRNA.

One of the major mechanisms whereby oxLDL contributes to endothelial dysfunction and atherosclerosis is the trans-activation of adhesion molecules, which mediate leukocyte adhesion to endothelial cells (Khan et al., 1995; Cominacini et al., 1997; Takei et al., 2001). Because it was observed that Neo-1 could be upregulated by oxLDL in vascular endothelial cells, we asked whether Neo-1 might play a role in leukocyte adhesion. To this end, endogenous Neo-1 was depleted by two independent pairs of siRNAs and knockdown efficiencies were verified by Western blotting (Figure 2A). Neo-1 depletion significantly suppressed the leukocyte adhesion to both EAhy926 cells and HAECs (Figure 2B). Similarly, the addition of an anti-Neo-1 blocking antibody diminished oxLDL-induced leukocyte adhesion to endothelial cells (Figure 2C). In contrast, Neo-1 over-expression, mediated by adenovirus mediated delivery of a Neo-1 vector (Figure 2A), did not appreciably influence leukocyte adhesion alone but enhanced leukocyte adhesion in the presence of oxLDL (Figure 2D).

**FIGURE 3:**
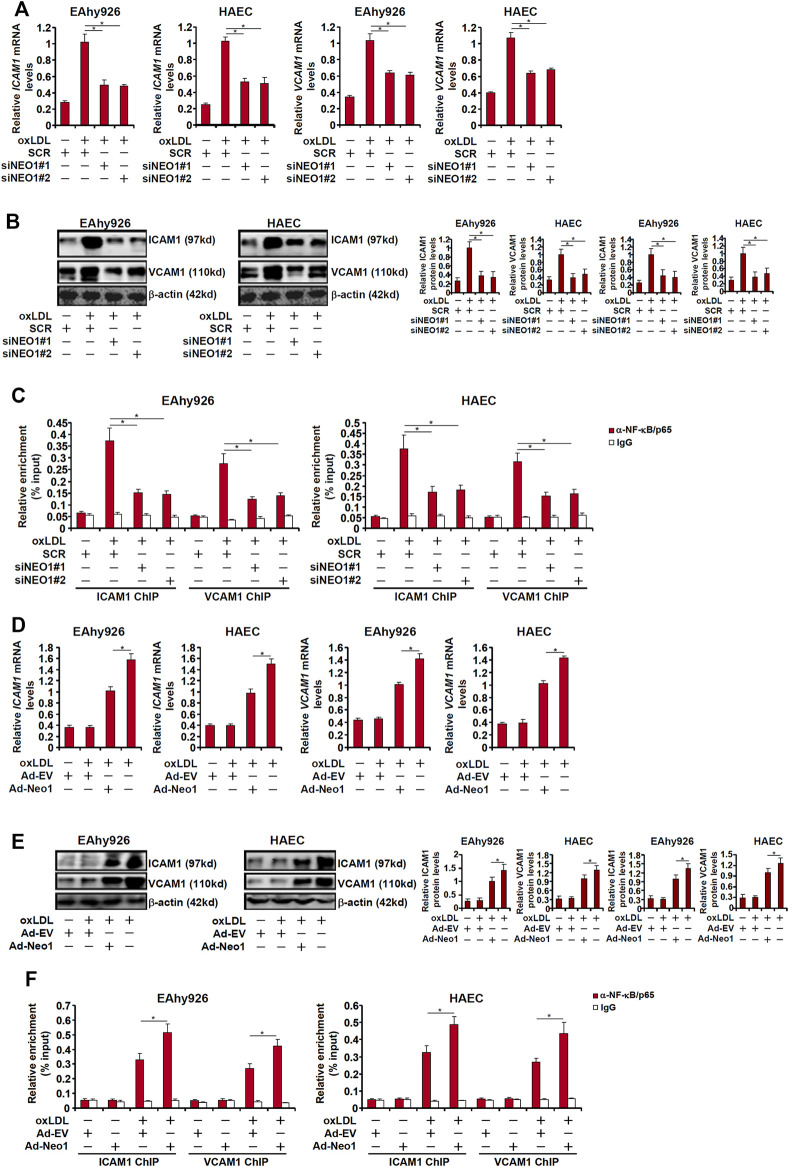
Neo-1 regulates expression of adhesion molecules by modulating *NF-κB* activity. **(A–C)** EAhy926 cells and HAECs were transfected with indicated siRNAs followed by treatment with oxLDL (50 μg/ml) for 24 h. Expression levels of adhesion molecules were examined by qPCR and Western blotting. ChIP assays were performed with anti-NF-κB or IgG. **(D–F)** EAhy926 cells and HAECs were transduced with adenovirus carrying a Neo-1 vector (Ad-Neo1) or control adenovirus (Ad-EV) followed by treatment with oxLDL (50 μg/ml) for 24 h. Expression levels of adhesion molecules were examined by qPCR and Western blotting. ChIP assays were performed with anti-NF-κB or IgG.

The pro-inflammatory transcription factor NF-κB is considered the master regulator for the trans-activation of a slew of adhesion molecules including ICAM1 and VCAM1 (Collins et al., 1995). Both ICAM1 expression and VCAM1 expression were upregulated by oxLDL treatment whereas Neo-1 knockdown attenuated induction of ICAM1 and VCAM1 (Figures 3A,B). Consistently, ChIP assay showed that oxLDL treatment strongly promoted the recruitment of NF-κB/p65 to the ICAM1 promoter and the VCAM1 promoter, which was weakened by Neo-1 knockdown (Figure 3C). On the contrary, Neo-1 over-expression enhanced the induction of ICAM1 expression and VCAM1 expression by oxLDL treatment (Figures 3D,E). The effect of Neo-1 over-expression on the adhesion molecule expression was likely attributable to the stronger association of NF-κB/p65 with target promoters (Figure 3F).

### Neogenin 1 Inhibition Attenuates Atherosclerosis in Mice

**FIGURE 4:**
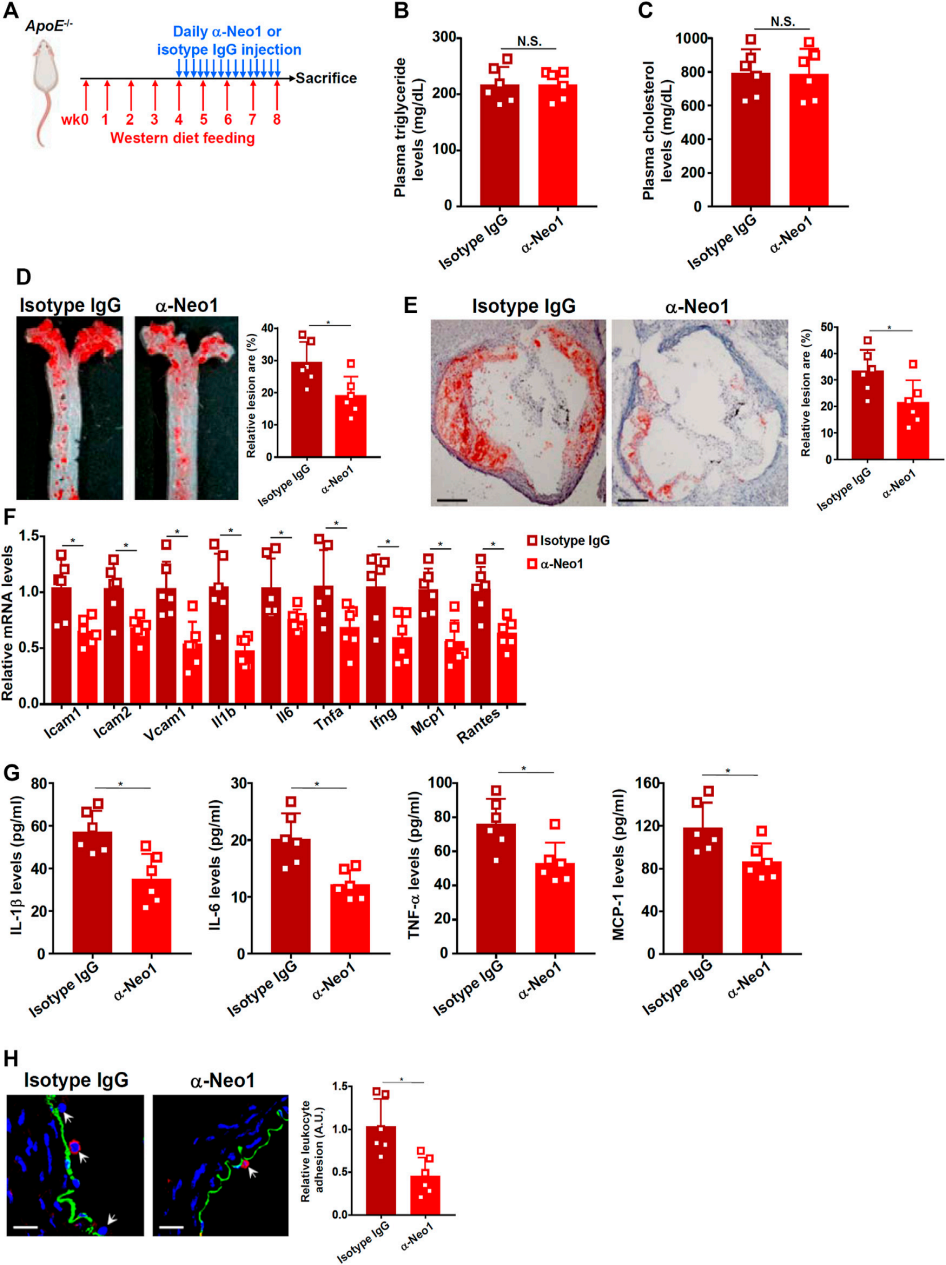
Neo-1 inhibition attenuates atherosclerosis in mice. **(A)** Scheme of animal protocol. **(B)** Plasma triglyceride levels. **(C)** Plasma cholesterol levels. **(D)** Oil red O staining of the thoracic aorta. **(E)** Oil red O staining of the aortic sinus. **(F)** Gene expression in aortic arteries was examined by qPCR. **(G)** Levels of pro-inflammatory mediators were examined by ELISA. **(H)** Infiltration of macrophages was examined by immunofluorescence staining. *N* = 6 mice for each group. Error bars represent SD (*p < .05, one-way ANOVA).

Next, we decided to extrapolate the finding that Neo-1 might be involved in endothelial dysfunction in a classic animal model in which *Apoe* ^−/−^ mice were fed a Western diet for 8 weeks to develop atherosclerotic lesions (Figure 4A). Blockade of endogenous Neo-1 was achieved by a blocking antibody that has been tested previously (Konig et al., 2012; Mirakaj et al., 2012; Schlegel et al., 2014); this antibody targets the extracellular domain of the Neo-1 protein (a.a.42-1033) thus disrupting the binding of Neo-1 to its ligands. Neo-1 inhibition did not significantly alter plasma triglyceride levels (Figure 4B) or plasma cholesterol levels (Figure 4C) suggesting that Neo-1 probably does not regulate hyperlipidemia. Oil red O staining of dissected aorta (Figure 4D) and aortic sinus (Figure 4E) indicated that compared to the isotype IgG injection, anti-Neo-1 injection significantly and markedly reduced atherosclerotic lesions. QPCR profiling showed that Neo-1 inhibition significantly down-regulated the expression of adhesion molecules and pro-inflammatory mediators including interleukin 1 beta (*Il1b*), interleukin 6 (*Il6*), tumor necrosis factor alpha (*Tnfa*), interferon gamma (*Ifng*), macrophage chemoattractive protein 1 (*Mcp1*), and regulated upon activation, normal T cell expressed and secreted (*Rantes*) in the aorta (Figure 4F). ELISA assays confirmed that protein levels of pro-inflammatory mediators were decreased by the administration of the Neo1-blocking antibody (Figure 4G). In addition, immunofluorescence staining confirmed that fewer leukocytes were detected to be adhered to the endothelium as result of Neo-1 inhibition (Figure 4H).

### cAMP Response Element Binding Protein 1 and Brg1-Associated Factor 47 are Essential for Transcriptional Activation of Neogenin 1 in Endothelial Cells

**FIGURE 5:**
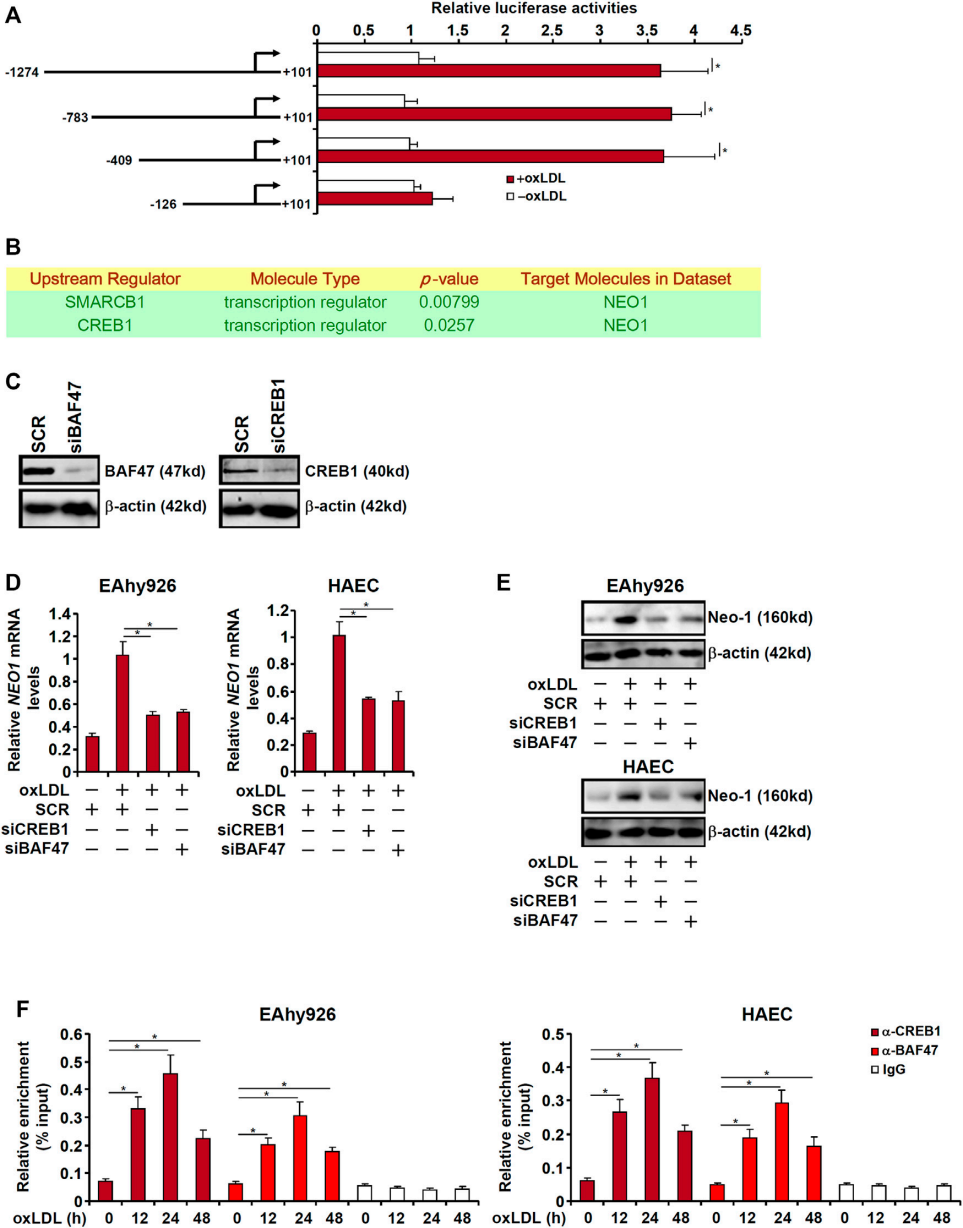
CREB1 and BAF47 are essential for transcriptional activation of Neo-1 in endothelial cells. **(A)** Neo-1 promoter-luciferase constructs were transfected into EAhy926 cells followed by treatment with oxLDL (50 μg/ml) for 24 h. Luciferase activities were normalized by protein concentration and GFP fluorescence. **(B)** Ingenuity pathway analysis. **(C)** EAhy926 cells were transfected with indicated siRNAs. Knockdown efficiencies were examined by Western. **(D,E)** EAhy926 cells and HAECs were transfected with indicated siRNAs followed by treatment with oxLDL (50 μg/ml) for 24 h. Neo-1 expression was examined by qPCR and Western. **(F)** EAhy926 cells and HAECs were treated with oxLDL (50 μg/ml) and harvested at indicated time points. ChIP assays were performed with anti-CREB1, anti-BAF47, or IgG.

The next set of experiments was designed to explore the mechanism underlying upregulation of Neo-1 expression by oxLDL in endothelial cells. First, full-length (−1274/101) and truncated (−783/+101, −409/+101, and −126/+101) Neo-1 promoter-luciferase fusion constructs were transfected into EAhy926 cells followed by oxLDL treatment. As shown in Figure 5A, oxLDL treatment robustly augmented the activities of the longer Neo-1 promoters but not the shortest Neo-1 promoter indicating that oxLDL likely regulates Neo-1 expression at the transcriptional level and that a potential response element might reside between −409 and −126 relative to the transcription start site. Ingenuity pathway analysis (IPA) revealed cAMP response element binding protein 1 (CREB1) and Brahma-related protein associated factor 47 (BAF47, encoded by *SMARCB1*) as the upstream transcriptional regulators of Neo-1 (Figure 5B). Knockdown of endogenous CREB1 and BAF47 with siRNAs (Figure 5C) suppressed the upregulation of Neo-1 expression by oxLDL stimulation in both types of endothelial cells (Figures 5D,E). More important, ChIP assay showed that oxLDL promoted the recruitment of both CREB1 and BAF47 to the Neo-1 promoter in a kinetics similar to that of Neo-1 induction (Figure 5F).

### cAMP Response Element Binding Protein 1 Interacts With and Recruits Brg1-Associated Factor 47 to Activate Neogenin 1 Transcription

**FIGURE 6:**
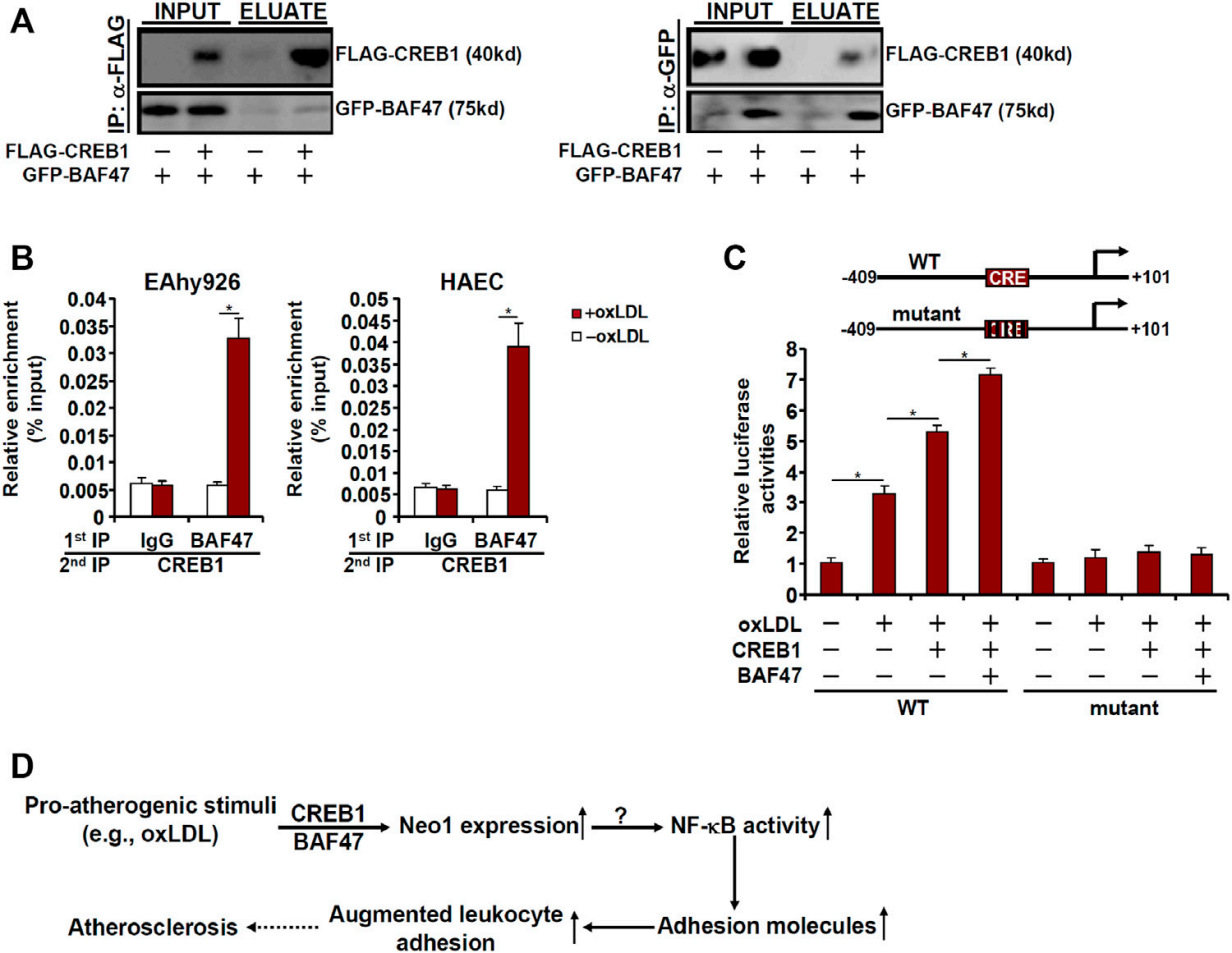
CREB1 interacts with and recruits BAF47 to activate Neo-1 transcription. **(A)** HEK293 cells were transfected with FLAG-tagged CREB1 and GFP-tagged BAF47 as indicated. Immunoprecipitation was performed with anti-FLAG or anti-GFP. **(B)** EAhy926 cells and HAECs were treated with or without with oxLDL (50 μg/ml) for 24 h. Re-ChIP assay was performed with indicated antibodies. **(C)** Wild type or mutant Neo1 promoter-luciferase construct (−409/+101) was transfected into EAhy926 cells with indicated expression constructs followed by treatment with oxLDL (50 μg/ml) for 24 h. Luciferase activities were normalized by protein concentration and GFP fluorescence. **(D)** A schematic model.

Because BAF47 is a transcriptional co-factor without a DNA binding domain that recognizes and binds to specific DNA sequences, we speculated that CREB1 might interact with BAF47 to cooperatively regulate Neo-1 transcription. When FLAG-tagged CREB1 and GFP-tagged BAF47 were co-transfected into HEK923 cells, an anti-FLAG antibody immunoprecipitated both CREB1 and BAF47 whereas an anti-GFP antibody simultaneously pulled-down both BAF47 and CREB1 suggesting that these two proteins could interact with each other (Figure 6A). Phosphorylation of CREB1 at serine 133 is key to its transcriptional activity (Johannessen et al., 2004). Of note, S133 phosphorylation mutation did not appear to influence the CREB1-BAF47 interaction. Interestingly, treatment with oxLDL enhanced the interaction between CREB1 and BAF47 (Supplementary Figure S3). More important, ChIP-on-ChIP (Re-ChIP) experiments demonstrated that oxLDL treatment stimulated the formation of a BAF47-CREB1 complex on the Neo-1 promoter (Figure 6B). In addition, reporter assay showed that co-expression of CREB1 and BAF47 enhanced the induction of the Neo-1 promoter activity by oxLDL. The synergistic effect between CREB1 and BAF47 on the Neo-1 promoter was not influenced by CREB1 phosphorylation because the CREB1 S133A mutant was able to cooperate with BAF47 to activate the Neo-1 promoter as potently as the wild type CREB1 (Supplementary Figure S4). However, when the putative CREB1 binding site was mutated within the Neo-1 promoter, neither CREB1 alone nor co-expression of CREB1 and BAF47 lost the ability to influence the Neo-1 promoter activity (Figure 6C).

## Discussion

Vascular inflammation is considered the pathogenic cornerstone of atherosclerosis. Adhesion of circulating leukocytes to the vascular endothelium triggers and perpetuates the inflammatory response. Here we describe a novel transcriptional pathway that connects Neo-1 activation to leukocyte adhesion in endothelial cells and potentially atherogenesis (Figure 6D).

Despite the observation that Neo-1 inhibition by a blocking antibody dampens atherogenesis in mice (Figure 4), several lingering issues deserve further attention. First, we focused on the regulation of Neo-1 by oxLDL in vascular endothelial cells. However, the possibility that Neo-1 upregulation by oxLDL in vascular smooth muscle cells (VSMCs) or macrophages may similarly contribute to atherogenesis and thus offer explanation to the observed phenotype cannot be ruled out. Hadi et al. (2018) have recently reported that activation of Neo-1 in VSMCs causes persistent stimulation of metalloproteinase 3 (MMP3) and consequently aberrant degradation of extracellular matrix leading to the pathogenesis of abdominal aortic aneurysm. Because phenotypic switch of VSMCs is a pathophysiological process shared by atherosclerosis and aneurysm (Chakraborty et al., 2021), it is plausible to speculate that Neo-1 might be upregulated by oxLDL in VSMCs and steer VSMCs to switch from a contractile phenotype to a pro-atherogenic phenotype. Alternatively, several reports have suggested that Neo-1 in the myelocytic compartment can potentially drive a pro-inflammatory response in different tissues (Konig et al., 2012; Mirakaj et al., 2012; Schlegel et al., 2014; Schlegel et al., 2019). Gulati et al. (2019) using low-input RNA-seq technique, have shown that Neo-1 positivity in myelocytic cells is associated with a pro-inflammatory signature of gene expression. In addition, motif enrichment analysis reveals that Neo-1 specifically enhances NF-κB activity (Gulati et al., 2019), which is in agreement with our data that Neo-1 critically regulates NF-κB activity in endothelial cells (Figure 3). These observations combined appear to suggest that Neo-1 might promote vascular inflammation and hence atherogenesis by skewing the phenotype of macrophages. Second, we relied on the induction of adhesion molecules and leukocyte adhesion as a readout to evaluate the effect of Neo-1 on endothelial dysfunction. Other aspects of endothelial deregulation in the context of atherogenesis should also be considered. For instance, aberrant neovascularization, or formation of new capillaries by endothelial cells, within the atherosclerotic plaque is observed in humans and model animals (Kwon et al., 1998; Moreno et al., 2006). In contrast, inhibition of aberrant angiogenesis can cause regression of atherosclerosis (Moulton et al., 1999; Gossl et al., 2009). Of interest, several independent reports demonstrate that Neo-1 activation elicits strong angiogenic response in endothelial cells (Park et al., 2004; Prieto et al., 2017; Yao et al., 2020). Therefore, attenuation of atherosclerosis by Neo-1 inhibition could be attributed to the suppression of aberrant neovascularization. Third, it is not clear at this point the signaling cascade that mediates the pro-atherogenic effect of Neo-1 in endothelial cells. Typically, Neo-1 signaling can be activated by one of the netrins (e.g., netrin 1). Indeed, netrin 1 deficiency has been shown to attenuate atherosclerosis in *Ldlr* ^−/−^ mice likely through evicting macrophages from the plaque and reining in chronic inflammation (van Gils et al., 2012). Alternatively, the protein structure of Neo-1 shares high degree of resemblance to that of pattern recognition receptors (PRRs); both possess several tandem immunoglobulin (Ig)-like domains and fibronectin type III domains (FnIII) (Wilson and Key, 2007). Because oxLDL can bind to and activate several different types of PRRs, including scavenger receptor (MSR1) and CD36, it is tempting to propose that Neo-1 could be directly bound and activated by oxLDL in endothelial cells to provoke a pro-atherogenic response. These unsolved issues clearly deserve further attention in the future.

We show here that CREB1 is necessary for oxLDL induced trans-activation of Neo-1 in endothelial cells by interacting with and recruiting BAF47. The pathophysiological relevance of this finding, however, remains to be determined. On the one hand, CREB1 down-regulation is observed in the vessels isolated from the atherosclerotic mice compared to the normal mice (Schauer et al., 2010). On the other hand, activation of the pro-inflammatory cytokine IL-17 by CREB1 is directly responsible for macrophages accumulation and the ensuing inflammation in the atherosclerotic plaque in mice (Kotla et al., 2013). Equally ambiguous is the role of CREB1 plays in endothelial homeostasis. A wealth of data seems to suggest that CREB1 deletion in endothelial cells may lead to increased inflammatory response and disrupted barrier function (Chava et al., 2012; Xiong et al., 2020). In contrast, CREB1 can promote leukocyte adhesion by directly binding to and activating the transcription of ICAM1 in human umbilical endothelial cells (Hadad et al., 2011). This apparent discrepancy likely alludes to the cell-type and context specific effects of CREB1 in atherogenesis. Future studies should exploit spatiotemporally controlled CREB1 transgenic animal models to carefully delineate the role of CREB1 in atherosclerosis.

In conclusion, our data unveil a previously unrecognized role for the CREB1-BAF47-Neo1 axis in regulating endothelial dysfunction that might potentially contribute to atherosclerosis. Additional functional and mechanistic studies are warranted to further validate the impact of this axis *in vivo* so that novel therapeutic solutions derived from this study can be devised in the intervention of coronary heart disease.

## Data Availability

The original contributions presented in the study are included in the article/Supplementary Material, further inquiries can be directed to the corresponding authors.
