# Supplementary material for: Upregulation of Neogenin-1 by a CREB1-BAF47 Complex in Vascular Endothelial Cells is Implicated in Atherogenesis
Source: Front Cell Dev Biol. 2022 Feb 3;10:803029. doi: 10.3389/fcell.2022.803029 (PMC8851423; doi:10.3389/fcell.2022.803029)
Supplement: Supplementary file 1 [file DataSheet1.docx]

**Li N et al: Up-regulation of neogenin-1 (Neo1) by a CREB1-BAF47 complex in vascular endothelial cells is implicated in atherogenesis**

**Online supplementary Figures**

**Fig.S1:** EAhy926 cells and HAECs were treated with oxLDL (50μg/ml) for 24h. Different cellular fractions were isolated. Neo1 levels were examined by Western blotting.

**Fig.S2:** HEK293 cells were transfected with FLAG-tagged CREB1 (WT or S133A) and GFP-tagged BAF47. Immunoprecipitation was performed with anti-FLAG.

**Fig.S3:** EAhy926 cells were treated with or without oxLDL (50μg/ml) for 24h. Immunoprecipitation was performed with anti-CREB1.

**Fig.S4:** Wild type or mutant Neo1 promoter-luciferase construct (-409/+101) was transfected into EAhy926 cells with indicated expression constructs followed by treatment with oxLDL (50μg/ml) for 24h. Luciferase activities were normalized by protein concentration and GFP fluorescence.
